# Mechanisms of Congenital Myasthenia Caused by Three Mutations in the *COLQ* Gene

**DOI:** 10.3389/fped.2021.679342

**Published:** 2021-11-29

**Authors:** Xiaona Luo, Chunmei Wang, Longlong Lin, Fang Yuan, Simei Wang, Yilin Wang, Anqi Wang, Chao Wang, Shengnan Wu, Xiaoping Lan, Quanmei Xu, Rongrong Yin, Hongyi Cheng, Yuanfeng Zhang, Jiaming Xi, Jie Zhang, Xiaomin Sun, Jingbin Yan, Fanyi Zeng, Yucai Chen

**Affiliations:** ^1^Department of Neurology, Shanghai Children's Hospital, Shanghai JiaoTong University, Shanghai, China; ^2^National Health Commission (NHC) Key Laboratory of Medical Embryogenesis and Developmental Molecular Biology and Shanghai Key Laboratory of Embryo and Reproduction Engineering, Shanghai, China

**Keywords:** *COLQ* gene, CMS, splicing mutation, missense mutation, exon deletion

## Abstract

The gene encoding collagen like tail subunit of asymmetric acetylcholinesterase (COLQ) is responsible for the transcription of three strands of collagen of acetylcholinesterase, which is attached to the endplate of neuromuscular junctions. Mutations in the *COLQ* gene are inherited in an autosomal-recessive manner and can lead to type V congenital myasthenia syndrome (CMS), which manifests as decreased muscle strength at birth or shortly after birth, respiratory failure, restricted eye movements, drooping of eyelids, and difficulty swallowing. Here we reported three variants within *COLQ* in two unrelated children with CMS. An intronic variant (c.393+1G>A) and a novel missense variant (p.Q381P) were identified as compound heterozygous in a 13-month-old boy, with the parents being carriers of each. An intragenic deletion including exons 14 and 15 was found in a homozygous state in a 12-year-old boy. We studied the relative expression of the *COLQ* and *AChE* gene in the probands' families, performed three-dimensional protein structural analysis, and analyzed the conservation of the missense mutation c.1142A>C (p.Q381P). The splicing mutation c.393+1G>A was found to affect the normal splicing of *COLQ* exon 5, resulting in a 27-bp deletion. The missense mutation c.1142A>C (p.Q381P) was located in a conserved position in different species. We found that homozygous deletion of *COLQ* exons 14–15 resulted in a 241-bp deletion, which decreased the number of amino acids and caused a frameshift translation. *COLQ* expression was significantly lower in the probands than in the probands' parents and siblings, while *AChE* expression was significantly higher. Moreover, the mutations were found to cause significant differences in the predicted three-dimensional structure of the protein. The splicing mutation c.393+1G>A, missense mutation c.1A>C (p.Q381P), and *COLQ* exon 14–15 deletion could cause CMS.

## Introduction

Congenital myasthenic syndrome (CMS) is a group of diseases caused by inherited disorders of the neuromuscular junction (NMJ). The main clinical manifestations of CMS include decreased muscle strength at birth or shortly after birth, respiratory failure, restricted eye movements, drooping of eyelids, and difficulty swallowing. According to the location of synaptic junction lesions, CMS can be divided into preprotrusion, synaptic basement membrane, and postsynaptic protein lesions. Moreover, CMS can exhibit two genetic patterns, i.e., autosomal dominant and autosomal recessive, with the latter being more common. CMS-related mutations have been identified in more than 20 genes to date, including *CHRNE, CHRNA1, CHRNB1, CHRND, COLQ, DOK7, PLEC1, CHAT, MUSK, ALG2, ALG14, GFPT1, SCN4A, AGRN, BN1, DNM2, DPAGT1, LAMB2, LRP4, PREPL*, and *RAPSN* (1).

Mutations in the collagen like tail subunit of asymmetric acetylcholinesterase (*COLQ*) gene, which are typically autosomal recessive, account for fewer than 10% of all identified mutations in patients with CMS (1) and can result in type V CMS, also called endplate acetylcholinesterase deficiency. The *COLQ* gene is located on the short arm of chromosome 3 and is responsible for the transcription of three strands of collagen of acetylcholinesterase (AChE) attached to the endplate of NMJs. COLQ is responsible for anchoring AChE in the synaptic space of the NMJ; thus, AChE degrades acetylcholine (ACh) released from the presynaptic nerve endings into choline and acetate. The mammalian skeletal muscle AChE is present as three asymmetric homotetramers, each comprising four globular catalytic subunits. Heteromeric asymmetric forms are composed of one (A 4), two (A 8), or three (A 12) homotetramers attached to a structural subunit made of three COLQ strands (2) COLQ is encoded by 455 amino acids and contains four functional domains, including the N-terminal domain, which binds an AChE tetramer, the collagen domain, the proximal C-terminal domain responsible for the formation of the triple helix, and the distal C-terminal domain. The C-terminal domain interacts with MuSK, a muscle-specific tyrosine kinase receptor (3, 4). COLQ has important regulatory functions at the synapse, controlling ACh receptor clustering and synaptic gene expression through its interaction with MuSK (5). COLQ is synthesized by muscle cells and is strictly expressed at the NMJ in fast muscles, whereas COLQ expression in extrasynaptic domains in slow muscle cells is relatively low (6). COLQ acts to concentrate and anchor the enzyme within the synaptic basal lamina. Therefore, mutations in *COLQ* may prevent the tail subunit from linking to catalytic subunits or failure of insertion into the synaptic basal lamina.

To date, more than 30 *COLQ* gene mutations have been reported, including transcoding mutations, missense mutations, and splicing mutations, and most mutations truncate the protein distally in the proline-rich region attachment domain, resulting in downregulation of COLQ protein and its associated enzyme at the basal lamina and thereby leading to lack of ACh degradation. These changes then lead to prolongation of signaling between the nerve and muscle cells, which can damage the muscle cells and lead to muscle weakness. Mutations found at the N-terminal proline-rich domain prevent attachment of the AChE catalytic subunits to the collagenic tail, whereas mutations in the middle part of the protein may prevent trimerization of the collagenic tail (7). By contrast, mutations in the C-terminal domain may impair anchoring of the enzyme within the synaptic basal lamina (8, 9). However, the full spectrum of mutations in the COLQ gene leading to CMS has not yet been identified.

In this study, we evaluated the mechanisms through which *COLQ* gene mutations facilitate the pathogenesis of CMS.

## Materials and Methods

### Variant Detection

We collected venous blood samples from two patients suspected of having *COLQ* mutations after obtaining informed consent from the patients and/or their families. This study was approved by the Ethics Committee of Shanghai Children's Hospital, and all protocols complied with Chinese bioethics laws and the Declaration of Helsinki.

Genomic DNA was isolated from blood samples and sheared using a Covaris Ultra Sonicator. A DNA library was then constructed, and quality was assessed. The DNA library was sequenced on an Illumina HiSeq 2500 platform (Illumina, Inc., CA, USA), according to the manufacturer's instructions for 150-bp paired-end reads. Next, the Burrows-Wheeler alignment tool (version 0.7.15) was used to align paired-end reads based on the University of California Santa Cruz and National Center for Biotechnology Information human reference genome (hg19/GRCh37). Common variants were filtered out based on frequencies (minor allele frequency <0.05) in the Exome Aggregation Consortium (http://exac.broadinstitute.org), the Exome Sequencing Project (https://esp.gs.washington.edu), and 1,000 Genomes Project (http://www.1000genomes.org) databases after the identified variants were interpreted and filtered by Ingenuity Variant Analysis (Qiagen Inc., Germany).

Real-time quantitative polymerase chain reaction (qPCR) amplification based on the SYBR-Green method was performed for the replication/deletion sites of target genes using an ABI Q6 quantitative fluorescence PCR instrument. The amplification curve was analyzed using a QuantStudio Real-time System, and the location of repeat/missing information was determined through internal gene correction and through external reference calculation using the 2^−Δ*ΔCt*^ method.

### RNA Extraction and Reverse Transcription

0.25 mL Fresh Blood Using a Total RNA Was Extracted Extraction kit According to the Manufacturer's Instructions (Qiagen). After Extraction, the Concentration and Purity of Total RNA Were Detected Using a Nucleic Acid Protein Analyzer. Next, Total RNA Was Used as the Template, and Reverse RNA Transcription Was Carried out Using Prime Script Strand CDNA Synthesis Kit/RT Master Mix, According to the Manufacturer's Instructions. The Reaction System Was as Follows: Template RNA, 5 μL (≤ 500 ng); Primer Script RT Enzyme Mix, 2 μL; And RNase-Free Water, 3 μL. The Samples Were Incubated at 37°C for 15 min and Then 85°C for 5 s.

### qPCR Analysis of *COLQ* Gene Expression

Fluorescence qPCR was carried out using cDNA obtained from the blood of patients, their parents and sisters and the control peers with a real-time fluorescence qPCR kit (Takara Biomedical Technology), according to the manufacturer's instructions. The primers used to detect the expression of the *COLQ* gene in the blood were as follows: patient 1 *COLQ* forward primer, 5′-TTCCAATCTCAGCAGCCCTT-3′ (overlapped with exons 1–2) and reverse primer, 5′-TGGGAAACCCTTTTCTCCTTT-3′ (overlapped with exons 12–13; expected amplicon length: 518 bp); patient 2 *COLQ* forward primer, 5′-ACCTGCAGGACAACTTATAAT-3′ (overlapped with exons 12–13) and reverse primer, 5′-TCTCCATATGACCCAGGGAGAT-3′ (overlapped with exons 16–17; expected amplicon length: 504 bp). The primers used to detect the expression of the *AChE* gene in the blood were as follows: forward primer, 5′-GTTCTCCTTCGTGCCTGTGGTA-3′ and reverse primer, 5′- ATACGAGCCCTCATCCTTCACC-3′. The reaction system (20 μL) contained cDNA (2 μL), upstream primer F (10 μM; 0.4 μL), downstream primer R (10 μM; 0.4 μL), 2 × SYBR Premix Ex Taq (10 μL), and sterilized water (7.2 μL).

The reaction conditions for general PCR were as follows: predenaturation at 95°C for 5 min; 30 cycles of denaturation at 95°C for 45 s, renaturation at 60°C for 45 s, and extension at 72°C for 30 s; and a final extension at 72°C for 5 min. The reaction conditions for fluorescence qPCR were as follows: predenaturation at 95°C for 30 s; 40 cycles of denaturation at 95°C for 5 s and renaturation at 60°C for 20 s; and a final extension at 65°C for 15 s. Samples were evaluated in triplicate. Data were evaluated using the 2^−ΔΔCt^ method for calculation of relative gene expression. Average values of triplicate experiments are shown.

### Protein Structure Prediction Using the I-TASSER Server for Non-sense Mutations

We predicted the structure of the COLQ protein of missense mutation c.1142A>C and the wild protein. The I-TASSER Suite pipeline consisted of four general steps: threading template identification, iterative structure assembly simulation, model selection and refinement, and structure-based functional annotation. The server is available at http://zhanglab.ccmb.med.~umich.edu/I-TASSER/.

### Conserved Sequence Analysis

We analyzed locus 381 in the COLQ protein sequence for humans, Norway rats, house mice, domestic cats, cattle (*Bos taurus*), tropical clawed frogs, and horses to predict sequence conservation at this locus (**Figure 2C**).

## Results

### Clinical Data

#### Proband 1

Proband 1 came to our hospital at 13-month-old. He was the second child of non-consanguineous parents and was delivered at full-term. His birth weight was 3,650 g. The child was admitted to the hospital for treatment of pneumonia, respiratory failure, and congenital laryngeal chondromalacia several times after birth. At 3 months of age, the child underwent supraglottic plasty under a support laryngoscope with general anesthesia. He later suffered from chronic laryngitis, laryngeal obstruction, and pneumonia. The child had a congenital atrial septal defect and patent ductus arteriosus. At 4 months of age, the child still had difficulties holding up his head and developed double eyelid drooping. The symptoms were mild after waking up, but the drooping became more obvious before going to bed and was accompanied by strabismus of the right eye and a normal left eye. The child could hold up his head at 7 months of age and sit up without assistance at 14 months of age, but he could not push up his upper body or stand. He could stand and walk with assistance at 22 mouths of age.

The patient's anti-MuSK antibody levels were normal (<0.001 nM), but anti-ACh receptor antibody levels were higher than normal (0.73 nM). His muscle enzyme (CK and CK-MB) levels were normal, and electromyography revealed no sharp wave of fibrillation in the tested muscles of the upper and lower limbs. The MUP (motor unit potential) part of the tested muscles was wider, and heavy contraction collection was reduced. His nerve conduction velocity indicated that motor and sensory nerve conduction velocity and amplitude as well as motor nerve F wave latency were normal. Additionally, repeat electrical nerve stimulation indicated that the amplitude attenuation of the compound muscle action potentials (CMAP) amplitude of the innervated facial nerve muscle and the distal extremity muscle of the tested muscle exceeded the normal range when stimulated by low frequency, whereas the amplitude of the CMAP amplitude of the tested muscle did not increase significantly when stimulated by high frequency. The patient's sister was healthy.

At 17 months of age, 0.05 mg/kg salbutamol was given orally twice per day, and the dosage was increased to 0.1 mg/kg three times per day after 2 months. At 19 months of age, the child could walk while holding hands with an adult. At 20 months of age, he could walk for 7–8 steps without assistance, and by 22 months of age, he could walk unassisted.

#### Proband 2

Proband 2, an 11-year-old boy, presented with drooping double eyelids after birth, accompanied by limited abduction of the eyes and no morning or evening symptoms. His parents did not note the specific symptoms during early childhood. He could lift his head at 7 months, sit at 9 months, walk with assistance at 10 months, and stand at 18 months. However, at this age, he could not walk steadily and tended to fall. At about 3 years of age, he could walk stably but would tire easily. He was the third child of non-consanguineous parents and had been delivered naturally at full term. His birth weight was 3,800 g, and he had two healthy older sisters (ages 18 and 13 years). His symptoms were mild, and he was able to attend school independently.

The patient's anti-MuSK antibody levels were normal (<0.001 nM), as were his anti-ACh receptor antibody levels (0.44 nM). Neostigmine tests were normal, and muscle enzyme levels were normal. However, electromyography revealed extension of insertion potential in some tested muscles. The polyphase potential and irregular waves were significantly increased in light shrinkage MUP. Early recruitment was observed in heavy shrinkage. His nerve conduction velocity indicated that motor and sensory nerve conduction velocity and amplitude as well as motor nerve F wave latency were normal. Repeat electrical nerve stimulation indicated that the amplitude attenuation of the CMAP amplitude of the innervated facial nerve muscle and the distal extremity muscle of the tested muscle exceeded the normal range when stimulated by low frequency, whereas the amplitude of CMAP of the tested muscle did not increase significantly when stimulated by high frequency. When he was 11 years old, salbutamol was given orally twice per day. He was able to walk longer and his ptosis was improved at the age of 12.

### Variant Detection

#### Proband 1

**Figure 1:**
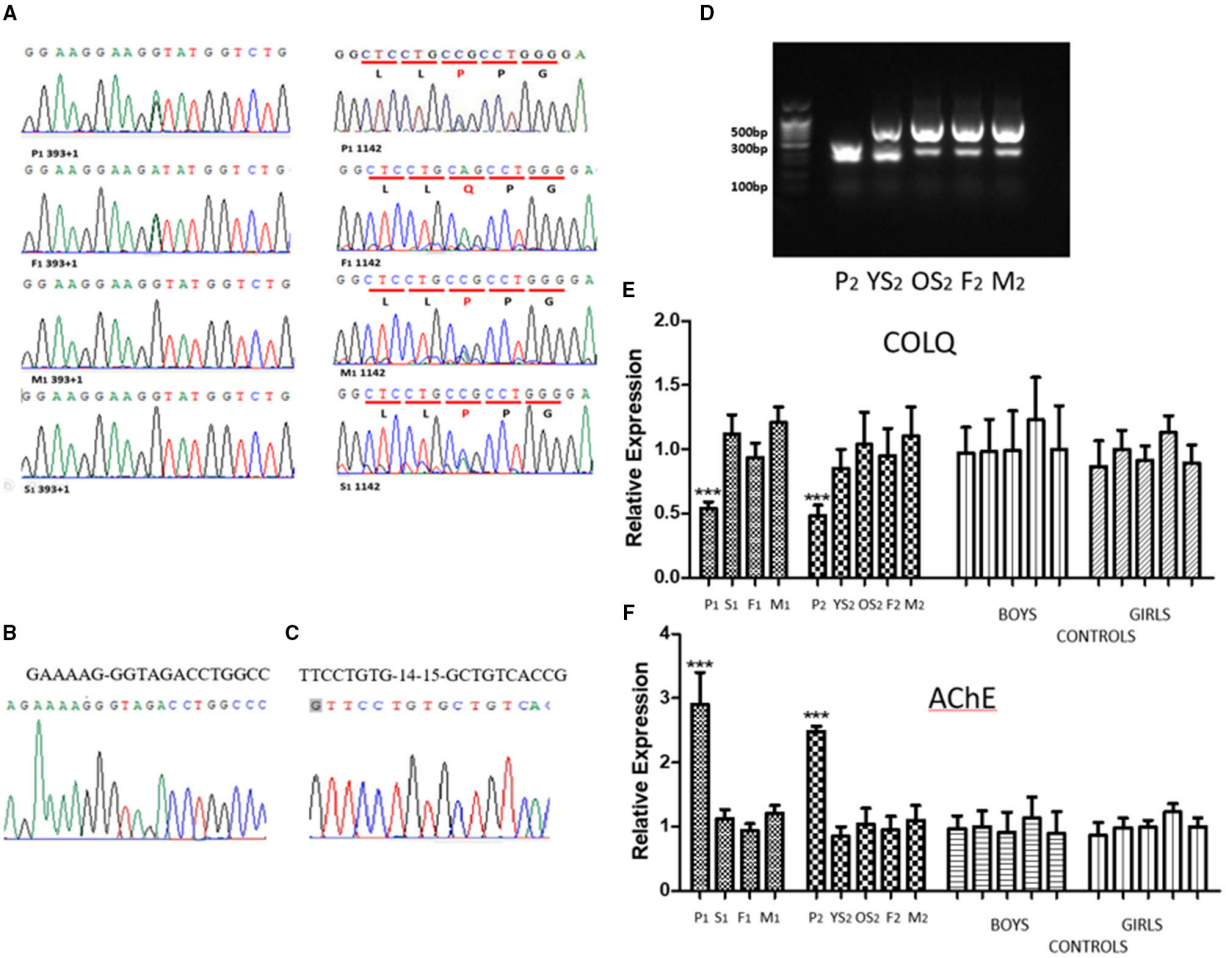
**(A)** The sequences of genomic DNA in the nuclear family of proband 1, with detection of a novel heterozygous splicing mutation in *COLQ* (c.393+1G>A) from the father and a missense mutation, c.1142A>C (p.Q381P) from the mother. His sister Inherited the missense mutation, c.1142A>C (p.Q381P) from mother. **(B)** The sequencing results of splicing mutation in *COLQ* (c.393+1G>A). **(C)** The sequencing results of deletion of *COLQ* exons 14–15. **(D)** PCR results of cDNA from the nuclear family of proband 2. Proband 2 (P2) produced only one truncated band, whereas both the parents (F2&M2) and sisters (P2YS& P2OS) had two identical truncated bands. **(E)** qPCR analysis. The expression level of the *COLQ* gene in proband 1 (P1) and proband 2 (P2) was significantly lower than that of the normal level. No decreased expression was observed in the proband's parents or sisters. **(F)** qPCR analysis. The expression level of the *AChE* gene in the two probands was significantly higher than that of the normal level. No decreased expression was observed in the proband's parents or sisters.

Sanger sequencing was performed for proband 1, his sister and parents. We identified a complex heterozygous mutation, i.e., the mutation c.393+1G>A from his father and the mutation c.1142A>C (p.Q381P) from his mother (Figure 1A).

#### Proband 2

Next-generation sequencing analysis revealed that proband 2 had a homozygous deletion of exons 14–15 in the *COLQ* gene and that his parents and two sisters were heterozygous carriers.

### RNA Extraction and Reverse Transcription

#### Proband 1

Proband 1 and his father produced a truncated band. However, no significant separation of PCR products was observed owing to the low number of missing bases. PCR products for the children and their parents were purified and sequenced. The results showed that the children and their father had a 27-bp deletion, resulting in deletion of nine amino acids. The sequencing results was provided in Figure 1B.

#### Proband 2

PCR products of proband 2 produced only one truncated band, whereas both the parents (F2&M2) and sisters (YS2 & OS2) had two identical truncated bands (Figure 1D). The truncated band was revealed a 224-bp deletion, resulting in deletion of many amino acids and a coding shift mutation, which caused premature termination of protein translation. The sequencing results was provided in Figure 1C.

### qPCR Analysis of *COLQ* and *AChE* Gene Expression

#### *COLQ* Gene Expression

The expression level of the *COLQ* gene in the two probands was significantly lower than that of the normal level. No decreased expression was observed in the proband's parents or sister (Figure 1E).

#### *AChE* Gene Expression

The expression level of the *AChE* gene in the two probands was significantly higher than that of the families and controls (Figure 1F).

### Changes in Protein Function and Structure

**Figure 2:**
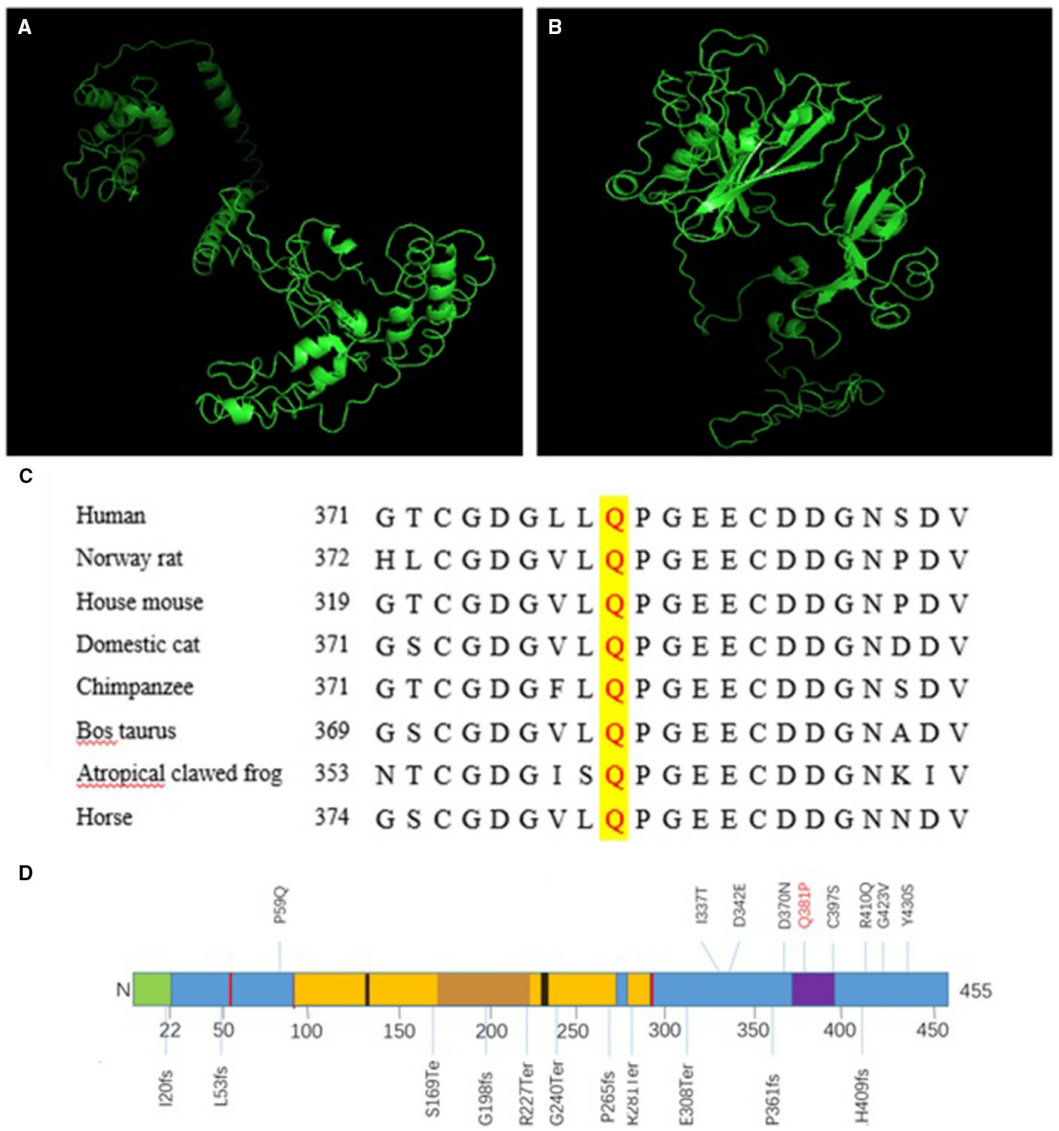
A Three-dimensional structure of COLQ. **(A)** Wild-type protein, **(B)** missense mutant [c.1142A>C (p.Q381P)]. **(C)** Conservation studies were conducted for c.1142A>C (p.Q381P) in humans, Norway rats, house mice, domestic cats, cattle (*Bos taurus*), tropical clawed frogs, and horses. **(D)** Primary structure and common mutations in the COLQ protein. Signal peptide: 1–22 (green); disulfide bonds: 51, 52, 93, 291, 293 (red); heparan sulfate proteoglycan binding: 130–133, 235–238 (black); collagen-like 1: 96–269 (yellow); *Myxococcus* cysteine-rich repeat: 373–397 (purple).

Next, we compared the three-dimensional structures of the wild type (Figure 2A) and missense mutated COLQ protein (Figure 2B) at http://zhanglab.ccmb.med. The missense mutated three-dimensional structure was different from the wild-type structure, and these changes were likely to affect the inherent stability of the protein as well as its protein-protein interactions.

### Conservation of Missense Mutations

Conservation studies were conducted for c.1142A>C (p.Q381P) in humans, Norway rats, house mice, domestic cats, cattle (*Bos taurus*), tropical clawed frogs, and horses (Figure 2C). These analyses suggested that glutamine was relatively stable. However, mutation to proline may cause changes in protein structure and function, thereby affecting protein function.

## Discussion

In this study, we report two cases of *COLQ* gene mutations, including a novel missense mutation c.1142A>C (p.Q381P) from the mother of proband 1; a novel splicing mutation c.393+1G>A from the father of proband 1; and exon 14–15 deletion, which had been reported previously in a Chinese patient (10), indicating the deletion of COLQ exons 14–15 might be a hot mutation in Han Chinese. Wang et al. also found that *COLQ* expression was significantly lower in the probands (10). Our results showed that AChE expression was significantly higher than that of the families and the controls, which concluded that COLQ mutations may affect the degradation of the esterase or functional abnormalities after the compensatory high expression. While it has been reported that median serum cholinesterase was significantly lower in patients with COLQ mutations than in controls (11). Due to insufficient sample size and the patients refused to provide venous blood samples again, the serum cholinesterase content could not be measured.

In the two cases described in this study, three mutations were identified. Proband 1 harbored a complex heterozygous mutation, in which the splicing mutation from the father was c.393+1G>A, leading to abnormal splicing and resulting in a 27-bp deletion corresponding to amino acids 123–131, which located in the region of heparan sulfate proteoglycan binding (Figure 2D). Thus, this mutation could prevent the formation of A12 and thereby affect the binding of COLQ to basement membrane polymucosaccharides. Accordingly, we inferred that the mutation resulted in a reduction in A12 formation and reduced COLQ binding to basement membrane polymucose, thereby affecting COLQ protein function. These findings were confirmed by analysis of COLQ expression in the probands vs. that in their families. Additionally, these results indicated that the mutation caused COLQ protein expression to decrease owing to premature termination codon (PTC) formation or synthesis of non-functional protein. Nonsense-mediated mRNA decay (NMD) blocks the expression of potentially toxic truncated proteins by identifying and degrading transcription products containing PTC. By predicting the three-dimensional structure of the post-shear protein, we found that the structure of the initial end of the post-shear protein N was significantly simplified. This change may have affected the binding of COLQ to AChE and the basement membrane, leading to the dysfunction of COLQ protein.

The missense mutation c.1142A>C caused the conversion of amino acid 381 from Q to P. This mutation was within a 10-cysteine-rich domain encompassing residues 375–451, which belonged to the C-terminal trimerization domain (Figure 2D). The glutamine residue at this position was conserved in humans, Norway rats, and most non-primate mammals (Figure 2C), and the change was predicted to impair anchoring of the enzyme within the synaptic basal lamina. By predicting the three-dimensional structure of the missense mutation, we found that the structure of the mutated protein was significantly different from that of the wild-type protein, further demonstrating that this site played important roles in the structural maintenance of COLQ protein and suggesting that mutation of this site could lead to abnormalities in the structure and function of the protein. Notably, COOH-terminal mutations in COLQ reduce binding of COLQ to MuSK and basement membrane extracts and decrease the activity of AChE bound to the basement membrane (4, 12).

In proband 2, homozygous deletion of exons 14–15 resulted in the deletion of 241 bp, yielding a code-shifting mutation, which terminated the synthesis of the peptide chain after an additional 26 amino acids. The final peptide chain was composed of 344 amino acids, resulting in the deletion of 111 amino acids in total. This caused decreased protein expression compared with that in the proband's parents and sisters. This region was located in the C-terminal trimerization domain, and the mutation could therefore prevent AChE from anchoring effectively. Moreover, PTC was produced due to a transcoding mutation, resulting in premature termination of peptide chain synthesis and the formation of a dysfunctional protein. NMD prevents the expression of truncated proteins with potentially toxic effects by recognizing and degrading transcriptional products containing PTC. Thus, both processes yield abnormal COLQ protein, which blocks AChE from anchoring normally and MuSK from binding normally. Notably, deletion of exons 14 and 15 in this patient could cause simplification of the C-terminal region of the protein. However, the clinical manifestations in this patient were mild, suggesting that there may be other mechanisms affecting the conduction of the nerve-muscle connector or that the mutation may affect the structure of the protein but allow the protein to retain some function. Importantly, in another study, mutation at the same site of the *COLQ* gene has been reported to result in different clinical manifestations, potentially because of nucleotide polymorphisms, RNA variable splicing, or environmental factors (13).

A model of CMS with AChE deficiency showed that both the embryonic γ-AChR subunit and the adult ε-AChR subunit in adult COLQ-deficient (COLQ-/-) mouse muscles were upregulated, resulting in the persistence of immature AChR and alterations in NMJ physiology. Downregulation of the mRNAs encoding these molecules was observed *in vitro* but not *in vivo*, suggesting the occurrence of compensatory mechanisms in muscles. In addition to myasthenia, COLQ-/- mice exhibit an atrophic phenotype, with fast muscles being more affected than slow muscles (6).

Patients with *COLQ* gene mutation develop the disease at a very early age, presenting with progressive muscle weakness. In the neonatal period, the main symptoms are dystonia, ptosis, eye paralysis, respiratory insufficiency, and even respiratory failure due to respiratory muscle weakness (14). Microcephaly has been reported in a few cases (15) and generally does not affect cognition (1). Movement development is difficult, and patients show delayed head support, sitting up, and walking, with a tendency to fall when walking. Electromyography shows double CMAP, slow pupil response to light, and no improvement or even deterioration of muscle weakness after the use of AChE inhibitors (16); these may be diagnostic clues for CMS caused by *COLQ* gene mutation (17). In this study, the clinical manifestations observed in the two patients were typical. The genetic diagnosis of proband 1 was confirmed, and neostigmine tests were not performed in order to avoid aggravating the disease. Gene sequencing was confirmed after negative results in neostigmine tests in proband 2. However, in our study, electromyography results did not show the typical double CMAP for either of the two patients.

As *COLQ* gene mutations account for fewer than 10% of all identified mutations in patients with CMS, the number of cases was small. Our experiment did have some shortcomings, such as small sample size and no *AChE* functional verification. We hope to encounter similar cases in future work and find the same site mutation in future articles to prove that the mutations can cause CMS.

*COLQ* mutation causes focal degeneration of the junction fold owing to AChR deletion. The junction contains denatured organelles, dilated vesicles, and apoptotic nuclei in electron micrographs (17). In a model of CMS with AChE deficiency, researchers showed that COLQ deficiency in mice was responsible for general muscle atrophy and defects in the formation of muscle fibers in fast muscles. Moreover, COLQ deficiency modified the composition of muscle fiber types (6). In a long-term follow-up study of 15 patients with *COLQ* gene mutations, researchers showed that esterase inhibitors, effort, puberty, or pregnancy could cause recurrence or aggravation of muscle weakness, which may be related to hormone levels. Some of these alterations were alleviated without intervention, whereas others required ephedrine or 3,4-diaminopyridine (3,4-DAP) treatment (6).

Therapeutic drugs used for the treatment of CMS include 3, 4-DAP, beta-ergic agonists, ephedrine, and albuterol. β2-Adrenergic receptor agonists that have been shown to promote muscle growth and strength (18). Additionally, analysis of salbutamol showed that this drug modified the NMJ in a mouse model of CMS and that the NMJs of salbutamol-treated mice showed significant alleviation of several postsynaptic morphological defects, including increased synaptic area, ACh receptor area and density, and extent of postjunctional folds. These effects occur primarily at the postsynaptic membrane and may lead to enhanced neuromuscular transmission (19). In another study, Ohno et al. found that a single intravenous administration of adeno-associated virus serotype 8-COLQ to *Colq*-/- mice rescued motor functions, synaptic transmission, and the ultrastructure of the NMJ. However, 48 weeks later, the motor functions of the treated mice were decreased to similar levels as those of wild-type mice. Thus, these findings established a novel potential approach for the treatment of patients with *COLQ* mutations (20).

Neostigmine tests have been shown to aggravate the symptoms of patients with CMS; thus, although neostigmine tests may be an important basis for differential diagnosis of other congenital muscle weaknesses, for infants and young children, the risks aggravating symptoms by neostigmine tests should be considered.

## Data Availability Statement

The original contributions presented in the study are included in the article/supplementary material, further inquiries can be directed to the corresponding author/s.

## Ethics Statement

The studies involving human participants were reviewed and approved by the Ethics Review Committee, Children's Hospital of Shanghai/Shanghai Children's Hospital, Shanghai Jiao Tong University. Written informed consent to participate in this study was provided by the participants' legal guardian/next of kin.

## Author Contributions

XLu, LL, and ChuW performed laboratory experiments and drafted the manuscript. FY, SWa, QX, RY, HC, YZ, and JX collected samples and analyzed clinical data. SWa, XLu, YW, JY, and FZ analyzed the genomic data. XLu and ChuW performed the formal analysis. LL, XLu, and FY assisted with the software and methodology. JZ, XS, and XLu performed the validation experiments. YC, SWu, and YZ edited the manuscript. Funds for the work were obtained by FY. All authors have approved the final manuscript.

## Funding

This work was supported by grants from the Shanghai Key Clinical Specialty Project (grant no. shslczdzk05705) and Key Disciplines of Top Priority in Shanghai (grant no. 2017ZZ02019).

## Conflict of Interest

The authors declare that the research was conducted in the absence of any commercial or financial relationships that could be construed as a potential conflict of interest.

## Publisher's Note

All claims expressed in this article are solely those of the authors and do not necessarily represent those of their affiliated organizations, or those of the publisher, the editors and the reviewers. Any product that may be evaluated in this article, or claim that may be made by its manufacturer, is not guaranteed or endorsed by the publisher.

## Abbreviations

COLQ: collagen like tail subunit of asymmetric acetylcholinesterase
CMS: congenital myasthenia syndrome
NMJ: neuromuscular junction
AChE: acetylcholinesterase.
